# Increased Brain Signal Variability Accompanies Lower Behavioral Variability in Development

**DOI:** 10.1371/journal.pcbi.1000106

**Published:** 2008-07-04

**Authors:** Anthony Randal McIntosh, Natasa Kovacevic, Roxane J. Itier

**Affiliations:** Rotman Research Institute of Baycrest Centre, University of Toronto, Toronto, Ontario, Canada; University College London, United Kingdom

## Abstract

As the brain matures, its responses become optimized. Behavioral measures show this through improved accuracy and decreased trial-to-trial variability. The question remains whether the supporting brain dynamics show a similar decrease in variability. We examined the relation between variability in single trial evoked electrical activity of the brain (measured with EEG) and performance of a face memory task in children (8–15 y) and young adults (20–33 y). Behaviorally, children showed slower, more variable response times (RT), and less accurate recognition than adults. However, brain signal variability increased with age, and showed strong negative correlations with intrasubject RT variability and positive correlations with accuracy. Thus, maturation appears to lead to a brain with greater functional variability, which is indicative of enhanced neural complexity. This variability may reflect a broader repertoire of metastable brain states and more fluid transitions among them that enable optimum responses. Our results suggest that the moment-to-moment variability in brain activity may be a critical index of the cognitive capacity of the brain.

## Author Summary

Intuitive notions of brain–behavior relationships would suggest that because children show more variability in behavior, their brains should also be more variable. We demonstrate that this is not the case. In measuring brain signal variability with EEG and behavior in a simple face recognition task, we found that brain signal variability increases in children from 8–15 y and is even higher in young adults. Importantly, we show that this increased brain variability correlates with reduced behavioral variability and more accurate performance. A brain that has more variability also has greater complexity and a greater capacity for information processing. The implication of our findings is that variability in brain signals, or what some would call noise, is actually a critical feature of brain function. For the brain to operate at an optimal level, a certain amount of internal noise is necessary. In a certain way it could be stated that a noisy brain is a healthy brain.

## Introduction

During neurodevelopment, behavioural performance tends to improve in terms of speed and accuracy. This improvement usually entails a decrease in trial-to-trial variability as performance approaches ceiling e.g. [1],[2]. A fundamental question is whether the neuronal dynamics that mediate behaviour show a similar decrease in variability. There are two arguments that furnish opposite predictions. The first is that neurodevelopmental trajectories will converge on optimal structure and dynamics, suggesting that trial-to-trial variations in evoked neuronal responses will decrease with age. This argument is particularly attractive in view of known neural pruning that accompanies normal brain maturation [3]. The second argument is that, if the computations underlying performance rest on adaptive, metastable brain dynamics [4], there may be an age-related increase in trial-to-trial variability. Functional variability represents a greater repertoire of metastable brain states and the more facile state transitions [5]. In this work, we disambiguated between these competing hypotheses by relating trial-to-trial variability in behavior with brain electrical activity across a sample of children and adults.

Neural systems can show a somewhat counterintuitive property, whereby optimal operations occur in the presence of a moderate amount of internal variability or noise [6]. For example, the phenomena of stochastic resonance describes how a simple nonlinear system can show an optimal signal-to-noise ratio with a moderate amount of noise, which enables the detection of weak periodic signals [7]–[9]. From cell channels to synapse to neural ensembles, noise seems to be an important parameter that shapes responsivity [10],[11]. It has been suggested that there may need to be a degree of physiological variability for the brain to adapt effectively to an uncertain environment [12]. While there are sophisticated computational models demonstrating the beneficial effects of noise for network dynamics [13],[14], the direct relation between neural variability and the behavior variability of the organism has been largely unexplored [15]–[17].

We sought to characterize the relation of neurophysiological variability and behavioral variability in maturation. Critically, our focus was not on variability of the signal across individuals within a group (interindividual), but rather the single-trial variability within an individual (intraindividual) [18]. Measures of single trial variability in electroencephalographic (EEG) signals within subjects were related to variability in response latency and accuracy. EEG signals were measured from children (ages 8–15 yrs, n = 55) and young adults (20–33 yrs, n = 24) during the performance of a face recognition task [19].

## Results

**Figure 1:**
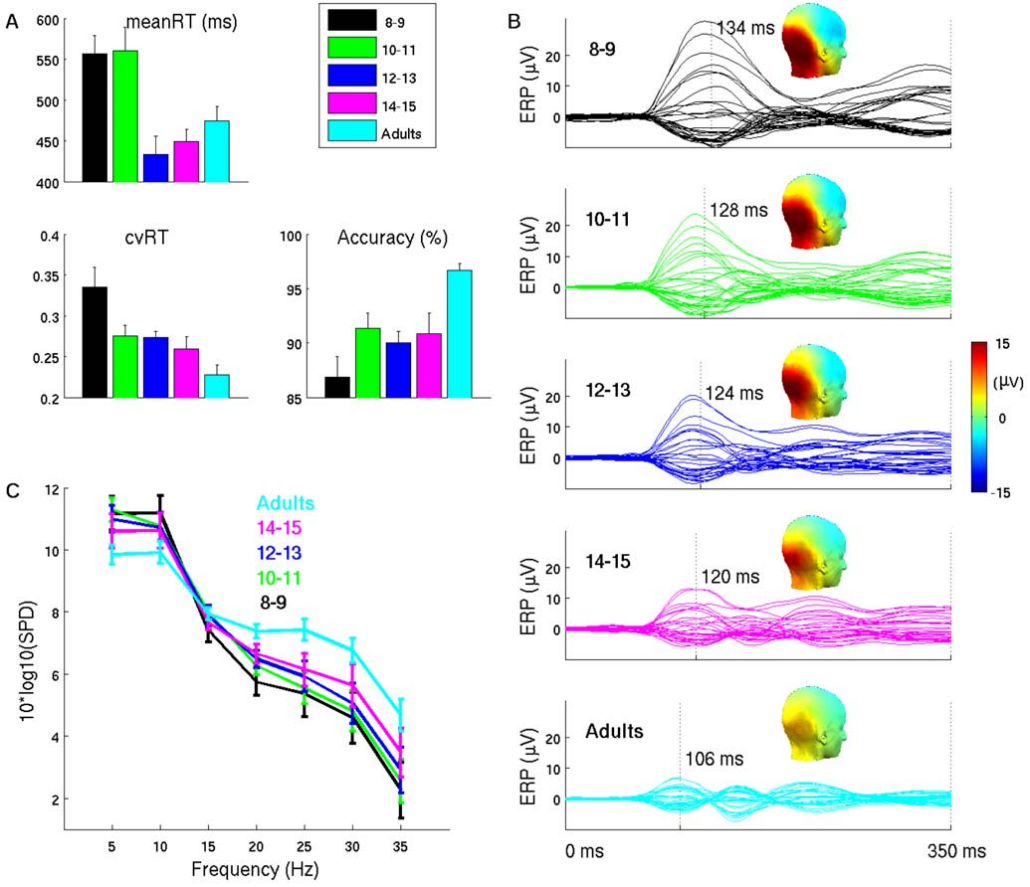
Behavior and EEG data by age. (A) Behavioral results for mean RT, coefficient of variation (cvRT) and accuracy (percent correct responses). Error bars indicate group standard errors. B) Waveforms for group average ERPs across all electrodes, together with corresponding P100 latencies (marked by vertical dashed line) and P100 scalp maps. C) Group average results for spectral power distribution (SPD) during baseline interval for electrode O2. Error bars indicate group standard errors. Similar pattern was present at all channels. With maturation, decreases were observed in lower frequencies (<10 Hz) combined with increase in higher frequencies (>10 Hz).

Behaviorally, all age groups showed high accuracy in the task, with adults near ceiling (Figure 1a). Recognition accuracy for children, while lower than for adults (one-way ANOVA, F (4, 74),  = 9.07, P<0.01), was well above chance. Mean reaction time (RT) was much slower for children 8–11 years and similar for children 12–15 years and adults (F (4, 74) = 6.65, P<0.01). Importantly, the coefficient of variation in reaction time (cvRT, standard deviation/mean RT within subject), which is an index of intra-individual variability, showed a gradual age-related decrease (F (4. 74) = 7.12, P<0.01). Though the standard deviation of RT (sdRT) showed the same trend as cvRT, because the standard deviation often scales with mean, we used cvRT which avoids this confound. Indeed the correlation between mean RT and sdRT was 0.63 and the correlation between mean RT and cvRT was 0.02. The correlation between sdRT and cvRT was 0.78.

The average stimulus evoked electrical potentials across the age groups (Figure 1b) showed a characteristic maturational change with greater amplitude but longer latency for a positive deflection peaking at about 100 ms post-stimulus (P100)[20],[21]. A second observation was that the subsequent deflections (N100, P200) are easily observable in adults and gradually emerge in children [19]. The bias in children towards higher amplitude, but slower, electrophysiological signals is paralleled by a differential distribution in prestimulus, or baseline, spectral power. Across age, there was a gradual reduction in low frequency spectral power and a relative increase in power at higher frequencies (Figure 1c, see supplementary material Text S1 for statistical analysis and Figures S1). The relative change in spectral power density presumably underlies the reduction in the latency of the evoked responses and its multicomponent nature [22],[23], where the lower frequency bias in children would yield slow and broad evoked potentials. The emergence of higher frequencies with maturation would both decrease the evoked response latency and allow additional deflections to emerge (i.e., N100, P200, etc.).

**Figure 2:**
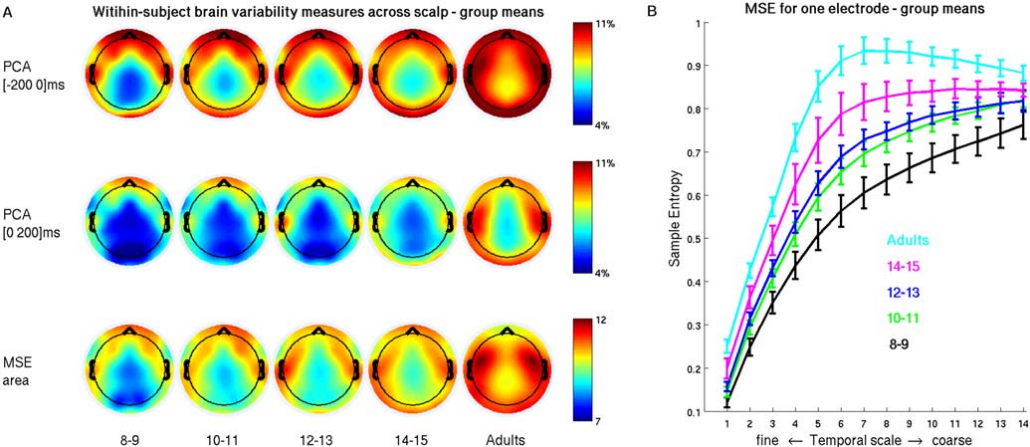
Within-subject brain variability measures across age groups. A) shows group mean results across the scalp for pre- and post-stimulus PCA dimensionality estimate of trial-to-trial variability (top two rows) and MSE area under the curve (bottom row). Scalp maps of group means were obtained by interpolating values from single channel group mean values. Gradual increase in all three brain variability measures across age groups is evident. PLS statistical analysis of each measure detected a pattern of linear increase, which was significant (p<<0 for all three measures) and was stably expressed (absolute bootstrap ratio >3.5) for most channels. B) shows group means for MSE estimates across temporal scales for channel O2, together with corresponding standard errors. Similar entropy curves were obtained for all channels and showed maturation-related increase in entropy at all scales. Given consistent age-related differences at all time scales, the area under the MSE curve was taken as a summary measure of maturational changes in entropy (e.g., (A), bottom row).

Two measures were used to evaluate brain signal variability. First, principal components analysis (PCA) was performed within each subject on their single trial EEG recordings. PCA identified the number of orthogonal dimensions, expressed as a proportion of the total possible, needed to express a certain amount of trial-to-trial variability (90% in the present case) for each channel. In a deterministic system with highly stereotyped responses, only a few dimensions are needed to capture most of the variability. To the extent that trial-to-trial recordings differ from one another, total variability increases, and hence PCA dimensionality increases. PCA dimensionality estimates for the 200-ms intervals pre- and post-stimulus onset increased across the age groups (Fig 2, a, b). Statistical analysis of PCA dimensionality with partial least squares [PLS, 24] confirmed a significant linear increase across age groups in dimensionality that was expressed stably across most EEG channels. In other words, adults showed the most variability in the measured brain signals.

A second aspect of signal variability is its temporal predictability, which can be measured using Multiscale Entropy [MSE 25]. MSE measures sample entropy [26],[27] of the signal at successively downsampled time series, with a scale of 1 being the original time series and scale *t* indicating a time series created by averaging *t* adjacent points. MSE assigns low values to both highly deterministic and completely random signals, making it an explicit measure of signal complexity [28].

MSE estimation applied to single trial data for each channel showed that sample entropy measures were highest for adults across all temporal scales and lowest for the youngest children, with the intermediate age groups falling along an ordinal trend (Fig 2b). Given that consistent age differences were observed at all time scales, the area under the MSE curve was computed for each subject to compare age-related differences. Multivariate statistical analysis with PLS confirmed a significant age-related increase in MSE that was expressed stably across most of the EEG channels (Fig 2a).

Taken together, the PCA and MSE measures indicate that, contrary to behavioral variability, brain variability increases with maturation. In some ways, this could be deduced from the group differences in the relative spectral density distribution, wherein one may expect signals that are dominated by low frequencies to show less variability than those with relatively stronger contributions from higher frequencies. For example, by increasing the relative magnitude of the Fourier coefficients for low frequencies in the adult EEG data, it is possible to get PCA dimensionality and MSE estimates similar to children (Figure S2). However, spectral density and variability are not completely interdependent, because jittering the phase of the Fourier coefficients, while maintaining their relative magnitude, has no impact on spectral density, but changes PCA dimensionality and MSE estimates (Figure S3). This is because PCA and MSE are sensitive to the dependencies within the signals that do not affect spectral density. Such sensitivities likely reflect transients in neural processing, and would be most evident in a system with enhanced capacity for signal processing and complexity.

The final and most important part of this investigation was to relate behavioral variability, brain variability, and maturation. We addressed these issues using PLS to analyze the correlations between our measures of dynamical variability (pre and post-PCA dimensionality and MSE) and their phenotypic correlates (RT-variability, accuracy, and age). We also include mean RT in the analysis to determine whether the correlation patterns we observed were specific to behavioral measures of variability, or to any metric showing a maturational change.

**Figure 3:**
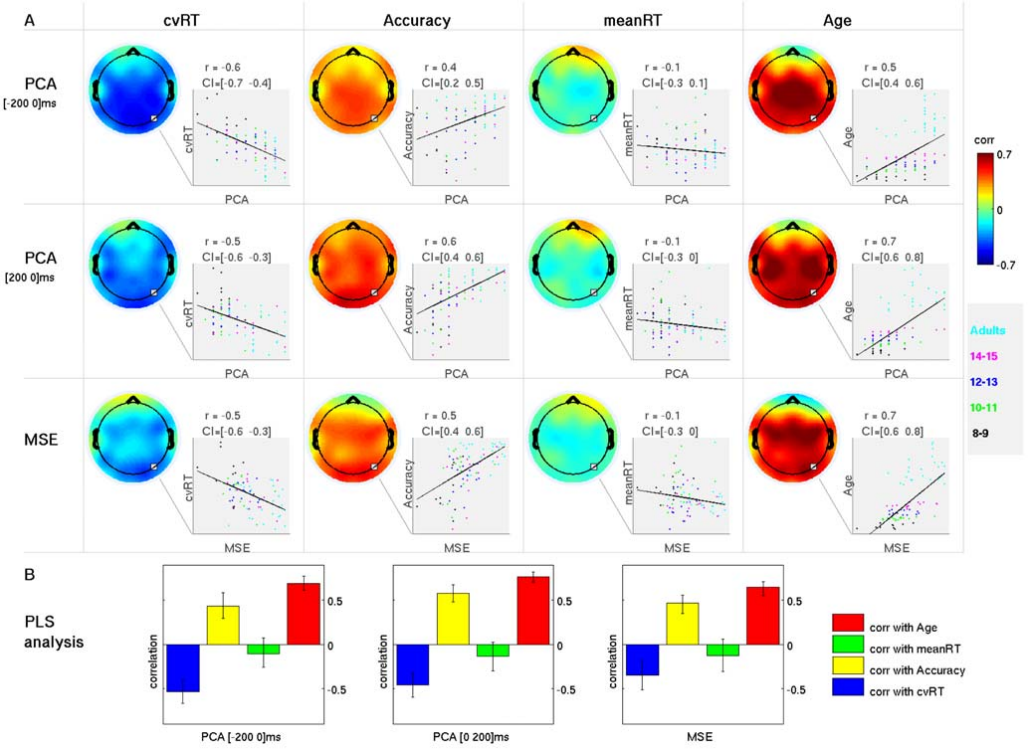
Within-subject brain variability in relation to behavior and chronological age. A) Correlations are arranged in a table where rows represent three within-subject brain variability measures (pre- and post- stimulus PCA and MSE) and columns represent behavioral measures (cvRT, accuracy and meanRT) and age. Each entry in the table shows a scalp map resulting from interpolated values from single channel correlations between given brain variability measure and given behavior or age. Unstable correlations (where the 95% CI included 0) were set to 0. Colormap corresponds to [−0.7 0.7] range of correlation values. Along with each scalp map of correlations, there is an inset showing a scatter plot representing subject measures for a single channel (electrode O2). Subjects are grouped by color according to age group membership. Estimated value of correlation ( r ) along with the associated 95% CI is given on top. B) Results of the statistical analysis of the observed correlations. For each brain measure, the partial least squares (PLS ) analysis detected one significant pattern of correlations (p = 0). The bar graph plots the global correlation of brain variability and each behavior measure or age (+/− bootstrap estimated standard error). As can be seen, for all three brain measures PLS detected similar global patterns of simultaneous negative correlation with cvRT, positive correlation with accuracy and age, and no stable correlation with mean RT. For each of the three within-subject brain variability measures, the bootstrap analysis of PLS confirmed that the correlation patterns were robustly expressed across most channels (data not shown).

Figure 3 shows the results of the analysis. Computed across all subjects, the correlation between behavioral variability (cvRT) and brain variability (PCA dimensionality and MSE) was negative and highly robust across most of the EEG channels (Figure 3a). The correlation for accuracy was a mirror image of the pattern for cvRT, showing a positive correlation with PCA and MSE estimates (Figure 3a). Mean RT, however, showed a much weaker, and statistically unreliable, correlation pattern with brain variability measures (Figure 3a). Finally, the correlation of chronological age and brain variability was very strong and positive across most of the scalp. The impressions derived from the visual inspection of Figure 3a were confirmed by the PLS analysis (Fig 3b). Measures of behavioral consistency (cvRT and accuracy) and chronological age showed stable correlations with the brain variability measured with PCA or MSE. Mean RT, however, did not. In other words, increased brain variability during maturation was associated with more stable and accurate behavior.

**Figure 4:**
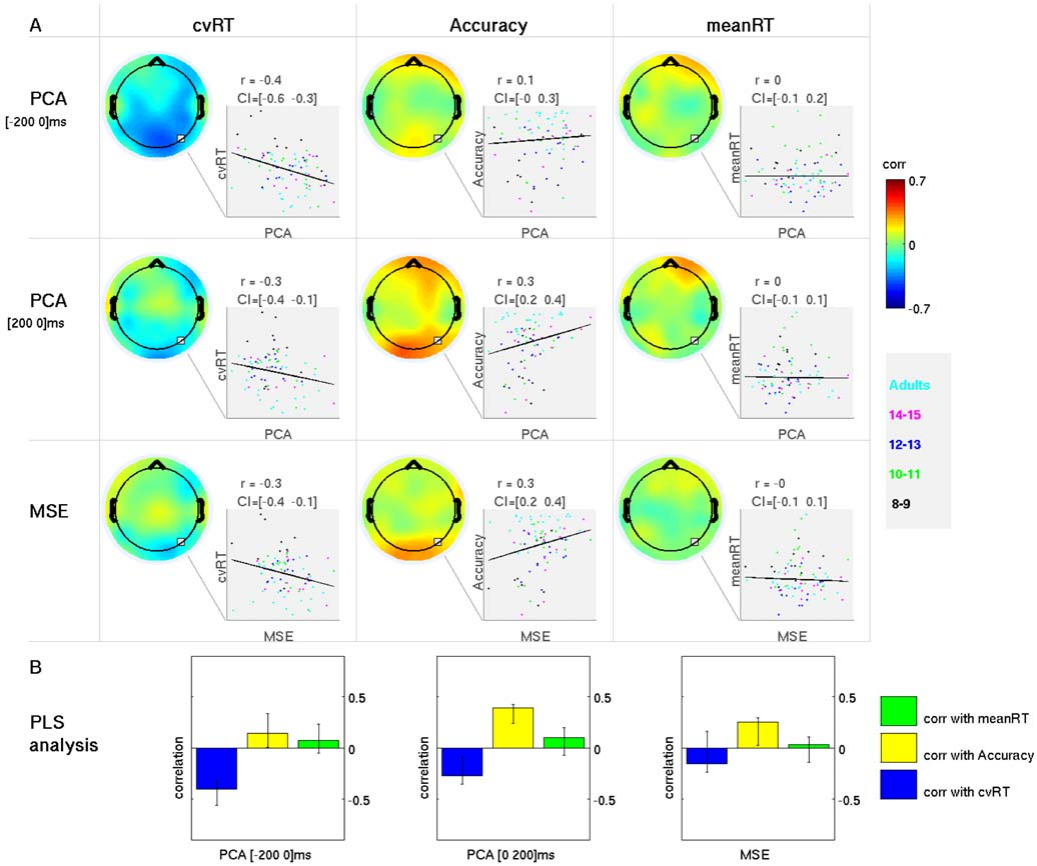
Relationship between within-subject brain variability and behavior after adjusting for age differences across individuals (age was regressed out from all measures). Color scale and legends are the same as for Figure 3. A) Correlations are arranged in a table where rows represent three within-subject brain variability measures (pre- and post- stimulus PCA and MSE) and columns represent three behavioral measures (cvRT, accuracy and mean RT). Each entry in the table shows a scalp map resulting from interpolated values from single channel correlations between given brain variability measure and given behavior or age. Unstable correlations (where the 95% CI included 0) were set to 0. Colormap corresponds to [−0.7 0.7] range of correlation values. Along with each scalp map of correlations, there is an inset showing a scatter plot representing subject measures for a single channel (electrode O2). Subjects are grouped by color according to age group membership. Estimated value of correlation (r) along with the associated 95% CI is given on top. B) Statistical analysis of brain-behavior correlations adjusted for age. PLS detected one significant pattern of correlations for pre- and post-stimulus PCA dimensionality estimation (p = 0.003, and p = 0.011), and one pattern for MSE that did not exceed conventional statistical thresholds (p = 0.131). Bootstrap analysis of the two significant patterns for PCA showed, however, that the patterns were unstable for most channels (data not shown).

The statement that the relation between behavioral variability and brain variability is mediated by maturation implies that if age differences were eliminated, the strong correlations seen in Figure 3 with cvRT and accuracy would be reduced. This turned out to be correct. We again used PLS to analyze the relation between the brain variability and behavior measures when chronological age was regressed out of both sets of measures. For cvRT and accuracy, the correlations for the full sample were reduced, and in the case of MSE, no longer statistically reliable (Figure 4). The effect was less dramatic when the analysis was performed on children only. Furthermore, the correlation between mean RT and brain variability remained nonsignificant after adjusting for chronological age for both the entire sample and the children. The age-adjustment process indicated that a large proportion of the relationship between behavioral consistency and brain variability was due to maturation, as measured by chronological age.

## Discussion

In contrast to behavioral variability, brain variability increases with maturation. With maturation comes differentiation and specialization of brain regions, but at the same time there is increased integration between distributed neuronal populations and establishment of new functional connections [29]. The change in balance between differentiation and specialization would produce more variability in on-going activity as the number of simultaneous processes possible at any given moment increases. Mature and integrated nervous systems generally have more prolonged and complicated neural transients [30]. Such transients are characteristic of a system with high neural complexity [31].

With the maturational increase in brain signal variability there is an increase in behavioral stability. Across the sample we studied, subjects with higher signal variability showed less variability in response latency (measured with cvRT) and greater performance accuracy. When the measures were adjusted for the chronological age, the relationship between brain and behavioral variability weakened, suggesting that a large part of the relationship represented a maturational effect. It is noteworthy that mean reaction time, which also showed a maturational change, did not significantly correlate with brain variability. It may be that other physiological factors are more important for the response speed change during maturation. By contrast, strong correlations with behavioral consistency indicates that cvRT and accuracy are likely tapping into aspects of the behavioral tuning which are more tightly related to the changes in brain complexity/variability.

The present results may seem at odds with the intuitive notion of behavior and brain variability, where one would expect that they go hand in hand. However, the results do make sense when the nonlinear dynamics of the nervous system are considered. Internal variability may be vital to enable the brain to parse weak and ambiguous incoming signals [10],[32],[33]. Variability can facilitate the exchange signals between neurons [34],[35], transitions in metastable systems [7], and the formation of functional networks [17],[36]. As the nervous system matures, physiological variability increases, which is captured by increases in complexity [37],[38], and the system can better adapt to its environment.

Maturational changes that have been reported in children's evoked potentials can also be related to increased brain signal complexity. Compared to adults, the average evoked responses in children tends to show higher amplitude and longer latency on early responses, and less well-defined later responses [20],[21],[39]. Spectral power distribution also changes with maturation, with a gradual reduction in low frequencies and an increase in higher frequencies [22],[40]. The relative change in spectral power density presumably underlies the reduction in the latency of the evoked responses and its multicomponent nature, where the lower frequency bias in children would yield slow and broad evoked potentials. The emergence of higher frequencies with maturation would both decrease the evoked response latency and allow additional deflections to emerge (i.e., N100, P200, etc.). Both the spectral power and evoked response changes would be expected given the maturational increase in complexity. The emergence of higher frequencies would reflect the enhanced local processing (segregation), whereas the multicomponent evoked response is thought to reflect reentrant interactions [41], suggesting enhanced integration.

In the age range of the children we studied the brain is in a state of structural and functional refinement [42],[43]. Myelination and neural pruning increase differentiation of information flow in the brain, enabling a shift from a system that responds in a slow and stimulus-locked manner, to one that responds more rapidly and where the internal variability reflects the parallel exploration of the functional repertoire before converging to an optimal response [16],[44]. In the case of normal development, the increased variability leads to a stabilization of behavior, increasing the cognitive repertoire of the system. One may postulate that internal variability would mature to some optimum level, based on both physiology and experience, but that further increases or decreases, coming from disease or damage would compromise behavioral stability. The suggestions derived from the present findings contribute to the growing evidence that internal dynamics are a key feature governing brain function [45]–[47].

## Materials and Methods

### Subjects

EEG recordings were collected from 24 adults and 55 children for a total of 79 subjects. Adults (18 females) ranged from 20 to 33 years of age. Children were divided into four age groups as follows: 8–9 years (n = 11, 3 females), 10–11 years (n = 16, 8 females), 12–13 years (n = 15, 8 females), and 14–15 years (n = 13, 6 females). Adult subjects and children, along with their parents, signed informed written consent. All subjects were healthy with no known cognitive or neurological disorders and had normal or corrected-to-normal vision. All children successfully completed two sub-tests of the WISC III (vocabulary and block design). The experimental procedure was approved by the French Comite Operationnel pour l'Ethique dans les Sciences de la Vie du CNRS.

### Apparatus and task

Continuous EEG was recorded (NeuroScan 4.1) on an EasyCap (10/10 system) containing 32 electrodes and Cz as reference, sampling rate of 500 Hz, a band-pass 0.1–100 Hz, and a gain of 500 (SynAmps). Subjects performed a rapid face recognition task. Each trial started with a presentation of a novel or familiar face for 500ms and subjects responded by pressing either a target or a non-target button depending on whether they recognized the face. Detailed description of the stimuli and the task are given in Itier & Taylor [19].

### Data preprocessing

Infraorbital electrodes for measuring eye movements were removed and an average reference was computed. The final number of electrodes was 31. Continuous EEG recordings were lowpass filtered at 40 Hz. Data were epoched and baselined into [−200 1200] ms epochs with a [−200 0] ms pre-stimulus baseline. Preliminary artifact removal was performed using independent component analysis (ICA) as implemented in EEGLAB software [48]. Trials contaminated with excessive amplitudes were removed first, then ICA decomposition was performed on the remaining concatenated trials and components carrying ocular and muscle artifacts were subtracted. The number of kept trials per subject was between 236 and 761, with an average of 529. For the signal variability estimation, it was important to have equal amounts of artifact-free data across subjects. We therefore introduced an additional trial selection step based on the total global field power (gfp), calculated as a sum of squared amplitudes across all electrodes and all time points for the trial duration. The 100 trials closest to the median for each subject were selected for further variability analysis. This selection criterion minimized potential presence of trials contaminated with high residual amplitude artifacts. This was particularly important for the convergence of multiscale entropy (MSE) algorithm (see below). It is critical to note that, with the exception of MSE, the results were similar when all trials that passed initial screening were analyzed.

### Behavioral measures

For each subject we calculated two response time related measures: mean response time (mean RT) and coefficient of variation of the response time (cvRT). The coefficient of variation of RT was calculated as the standard deviation divided by the mean RT within subject, and was taken as a measure of subject's behavioral variability. The scaling procedure in cvRT minimizes differences between groups that arise from differences in mean and standard deviations. Mean and cvRT were based on thresholded RTs (<1200 ms) and included both correct and incorrect responses. Exclusion of excessively long RTs as outliers enabled robust estimation of mean RT and cvRT. Because the trial selection focused on EEG signal regardless of the response, it is unlikely that such selection would have introduced any bias pertaining to the RT-related measures. Our results for mean RT are somewhat different than those presented in Itier & Taylor [19], where subject's mean calculation included only correct-response trials and employed no outlier thresholding. As a third behavioural measure, we used subject accuracy (percent correct responses), calculated from all recorded trials.

### Baseline spectral power distribution (baseline SPD)

Spectral power distribution of the baseline signal across single trials was calculated using Fast Fourier Transform (FFT). Considering the known age-related differences in global signal power, the signal was first normalized (mean = 0, standard deviation = 1) in order to calculate relative contributions of different frequency bands to the total spectral power. With 500 Hz sampling rate and 200 ms baseline signal this gave us 100 time points and 5 Hz frequency resolution.

### Principal Components Analysis (PCA) estimation of trial-to-trial signal variability

For each subject, PCA was used as an estimate for the dimensionality of the single trial EEG space. Subject's data was divided into channel specific matrices of single trial data with trials as rows and time points as columns. The dimensionality of each matrix was determined as a minimum number of principal components capturing 90% of the variance across trials. This number was further expressed as a percent of the total number of trials and was taken as a measure of trial-to-trial variability for a given channel. Two time intervals were analyzed: [−200 0]ms pre-stimulus and [0 200]ms of the post-stimulus signal. In this way, we obtained dimensionality estimates of the trial space for pre- and post-stimulus signals across the scalp. For example, average pre-stimulus PCA dimensionality estimate for electrode O2 within the adult group was 10.8. Given 100 trials, and hence 100 dimensions, this result means that for adults, 90% of the trial space signal variance occupies 10.8 dimensions.

### Multiscale Entropy estimation of temporal signal complexity

MSE was used to estimate entropy at different time scales. Full details of the MSE measure and its relevance for the analysis of signal complexity are given in Costa et al.,[49] and Costa et al., [28]. We first calculated single trial MSE using the algorithm available at www.physionet.org/physiotools/mse/ with parameter values m = 2, r = 0.5. The algorithm calculates sample entropy as a measure of regularity (predictability) of the signal at different scales. It consists of two procedures: 1) coarse-graining of the time series and 2) calculating sample entropy for each coarse-grained time series. For scale t, the coarse-grained time series is constructed by averaging the data points within non-overlapping windows of length t. This procedure can be viewed as a smoother version of decimation. Sample entropy of each coarse-grained time series measures its regularity by evaluating the appearance of repetitive patterns. The length of single trial time series was 700 time points corresponding to [−200 1200]ms epoch at 500 Hz sampling rate. For each subject, a channel specific MSE estimate was obtained as a mean across single trial entropy measures for scales 1–14. Entropy measures for scales >14 were not calculated because the corresponding coarse-grained (downsampled) time series were too short (<50 time points) for reliable sample entropy estimation.

### Partial least squares analysis

Statistical assessment of maturational trends in MSE and PCA was performed using partial least squares (PLS) for EEG data [24]. PLS was performed on data matrices consisting of subject and channel specific measures such that rows represented subjects within age groups. The columns of the data matrix were either the integrated measures for MSE or the PCA dimensionality estimation by channel. PLS data matrices were averaged within group and grand mean centered across all five age groups. The mean-centered matrices were then decomposed with singular value decomposition (SVD) to identify the strongest group differences and the corresponding scalp topography. For brain-behavior analyses, correlations were computed between each behavior measure and either the PCA or MSE measures across the entire sample. The four correlation “maps” (one each for cvRT, accuracy, mean RT, and chronological age) were then decomposed with SVD.

The statistical significance of the effects was assessed using permutation tests for the overall relationship between either age group and brain variability or brain and behavior. The reliability of the topographies was determined with bootstrap estimation of confidence intervals, using 500 bootstrap samples. For scalp topographies, the singular vector weights for each channel were divided by the bootstrap estimated standard error, giving a bootstrap ratio. This is similar to a z-score if the distribution of singular vector weights is Gaussian. Details of these statistical tests are described in [24],[50],[51].

At the univariate level, correlations involving chronological age, cvRT, accuracy, and mean RT (on the behavior side) were assessed for statistical reliability. MSE and pre- and post-stimulus PCA (on the brain side) were also assessed. The stability of the correlations was estimated across subjects using a bootstrapping procedure. This allowed us to calculate confidence intervals (CI) around the correlation levels. For each brain measure we generated 1000 random samples of subjects with replacement and calculated the corresponding correlation with a behavioral measure. The lower and upper 95th percentiles across bootstrap samples were derived giving us the 95% confidence interval. Correlations were considered reliable if the CI did not include zero.

For PCA trial space dimensionality estimation, the correlations across all 79 subjects were calculated for each channel. For MSE, we used area under the MSE curve (sum of entropy values across all scales) and calculated correlations with a behavioral measure for each channel. It should be emphasized that the inferential tests for the significance of these correlations were assessed with multivariate PLS. The channel-wise correlation analyses are an assessment of the reliability of the correlation patterns and are complementary to the multivariate PLS analysis.

## Supporting Information

Text S1 Supporting Text.
(0.03 MB DOC)

Figure S1 Statistical analysis of age related changes in spectral power distribution of the baseline signal (SPD). A) Group mean results for SPD for one channel (O2). Error bars indicate group standard errors. During maturation, one can observe a gradual increase in relative contributions from high frequencies (>10 Hz) and decrease in relative contributions from low frequencies (>10 Hz). B) PLS detected one significant pattern (p = 0) of linear changes related to maturation. C) Bootstrap analysis of pattern expressions across channels and frequencies. For each frequency and each channel, bootstrap ratios were thresholded (pattern was stably expressed if absolute bootstrap ratio exceeded 3.5). From the thresholded values for each frequency, a scalp map was interpolated across channels. The resulting bootstrap ratio maps indicate regions of stable expressions of the pattern from B), with red and blue regions corresponding to stable positive and negative expressions, respectively. Grey regions indicate regions of no stable expression. A stable pattern of increase in spectral contribution was observed at higher frequencies (> = 15 Hz). Inverted pattern, i.e., decrease in spectral contribution was observed at 5 Hz only. At 10 Hz, there was no stable pattern expression.
(0.49 MB EPS)

Figure S2 Modifying signal by biasing spectral power distribution towards lower frequencies. Relative increase in lower frequencies is paralleled by relative decrease in higher frequencies, while the total spectral power remains unchanged. This is shown in panel B. Panel A shows single trial time series of original and modified signals. Note the effect of smoothing due to the decrease in higher frequencies. Comparative results for PCA and MSE are shown in panels C and D, respectively. Error bars in the MSE graphs represent standard errors associated with mean MSE across single trials.
(0.57 MB EPS)

Figure S3 Modifying signal by randomizing phase while keeping power spectrum unchanged. Panel Ashows single trial time series of original and modified signals. Panel B shows average signal across trials. Note how typical ERP components like P1, N1 and P2 are missing in the modified signal because randomization procedure destroyed any phase relationship across trials. Comparative results for PCA and MSE are shown in panels C & D, respectively. Error bars in the MSE graphs represent standard errors associated with mean MSE across single trials.
(0.67 MB EPS)
