# Supplementary material for: Increased Brain Signal Variability Accompanies Lower Behavioral Variability in Development
Source: PLoS Comput Biol. 2008 Jul 4;4(7):e1000106. doi: 10.1371/journal.pcbi.1000106 (PMC2429973; doi:10.1371/journal.pcbi.1000106)
Supplement: Text S1 — Supporting Text. (0.03 MB DOC) [file pcbi.1000106.s001.doc]

Increased Brain Signal Variability Accompanies Lower Behavioral Variability in Development

Anthony Randal McIntosh, Natasa Kovacevic, Roxane J. Itier

**Supplementary Material**

***Statistical analysis of age-related changes in spectral power distribution (SPD) and its relationship with behavior and chronological age***

As for the three within-subject brain variability measures (pre- and post-stimulus PCA, MSE), we performed multivariate statistical analysis of SPD using PLS. In the case of SPD, we calculated relative contributions at frequencies 5Hz, 10Hz, etc. to 35Hz to the total spectral power. Each frequency band was entered as a separate variable into analysis. PLS results of age-related changes in SPD are shown in Fig S1. PLS detected a linear pattern of maturation-related changes. A stable pattern of increase in spectral contribution was observed at higher frequencies (>=15Hz). An inverted pattern, i.e., a decrease in spectral contribution was observed at 5Hz only. At 10Hz, there was no robust pattern expression.

***Relationship between brain signal measures (SPD, PCA, and MSE)***

The three measures used in this paper address different aspects of signal variability. PCA gives us an idea of how many distinct time courses the brain produces. MSE measures temporal dependencies of the signal at different time scales. SPD is a measure of relative contributions from different frequency bands, and may be related to both the PCA and MSE estimates. In one set of simulations, we investigated this relationship. We started with one set of 100 trials from an arbitrary adult subject and channel. Each trial time course was transformed using FFT. We then modified spectral power distribution of the signal by increasing magnitudes of Fourier coefficients for frequencies < 12Hz and decreasing magnitudes of coefficients for frequencies >=12Hz. The modified Fourier representation was subsequently transformed back into the time domain using Inverse Fast Fourier Transform (IFFT). The resulting modified time course was adjusted to have the same mean and standard deviation as the original signal. The purpose of this modification was to redistribute the spectral power of the signal by giving more relative power to lower frequencies, while keeping total power and phase unchanged. We then calculated MSE and PCA measures of original and modified signals. The comparison between the original and modified signals in terms of MSE and PCA measures is shown in Fig S2. It shows that the introduced increase in relative power at lower frequencies resulted in a decrease of PCA and MSE, resembling the way the signal of children differs from that of the adults.

PCA and MSE are not, however, solely dependent on SPD. To show this, we ran a second set of simulations in which phase was randomly assigned, while keeping SPD the same. Using the same 100 trials as before, we calculated FFT of each trial. We then modified Fourier coefficients by assigning random phases, while keeping magnitudes the same. The time course of the modified signal was obtained using IFFT. We hypothesized that randomization of the phase would destroy any temporal dependencies in the signal and phase similarities across trials. Indeed, this was confirmed by comparing MSE and PCA measures as shown in Fig S3. This simulation suggests that the PCA and MSE measures are sensitive to non-stationarities in the physiological signal that are not captured by SPD. Such non-stationarities may reflect transients in neural processing. These simple simulations suggest that the three measures provide complementary, rather than redundant, information on the brain signal complexity.

Captions for Supplementary figures

Figure S1. Statistical analysis of age-related changes in spectral power distribution (SPD) of the baseline signal. A) Group mean results for SPD for one channel (O2). Error bars indicate group standard errors. During maturation, one can observe a gradual increase in relative contributions from high frequencies (>10Hz) and decrease in relative contributions from low frequencies (>10Hz). B) PLS detected one significant pattern (p=0) of linear changes related to maturation. C) Bootstrap analysis of pattern expressions across channels and frequencies. For each frequency and each channel, bootstrap ratios were thresholded (pattern was stably expressed if absolute bootstrap ratio exceeded 3.5). From the thresholded values for each frequency, a scalp map was interpolated across channels. The resulting bootstrap ratio maps indicate regions of stable expressions of the pattern from B), with red and blue regions corresponding to stable positive and negative expressions, respectively. Grey regions indicate regions of no stable expression. A stable pattern of increase in spectral contribution was observed at higher frequencies (>=15Hz). An inverted pattern, i.e., a decrease in spectral contribution was observed at 5Hz only. At 10Hz, there was no stable pattern expression.

Figure S2. Modifying signal by biasing spectral power distribution towards lower frequencies. Relative increase in lower frequencies is paralleled by relative decrease in higher frequencies, while the total spectral power remains unchanged. This is shown in top right panel. Top left panel shows single trial time series of original and modified signals. Note the effect of smoothing due to the decrease in higher frequencies. Comparative results for PCA and MSE are shown in bottom left and right panels, respectively. Error bars in the MSE graphs represent standard errors associated with mean MSE across single trials.

Figure S3. Modifying signal by randomizing phase while keeping power spectrum unchanged. Top left panel shows single trial time series of original and modified signals. Top right panel shows average signal across trials. Note how typical ERP components like P1, N1, and P2 are missing in the modified signal because randomization procedure destroyed any phase relationship across trials. Comparative results for PCA and MSE are shown in bottom left and right panels, respectively. Error bars in the MSE graphs represent standard errors associated with mean MSE across single trials.
